# Bacteria and Their Relations to Certain Diseases

**Published:** 1891-07

**Authors:** Charles Seth Evans

**Affiliations:** Cincinnati, O.


### BACTERIA AND THEIR RELATIONS TO CERTAIN DISEASES.[1]

By CHARLES SETH EVANS, B. S., M. D., Cincinnati, O.

Bacteria, microörganisms and germs are synonyms for a large family of extremely small, microscopical organisms. They belong, undoubtedly, to the vegetable kingdom, where they occupy the lowest position.

Structurally they are cells, and consist of a cell wall, said to contain cellulose, and cell contents; a nucleus is not known to exist, though certain varieties of bacteria do, under favorable circumstances, develop in the cell contents a body which possesses certain points of analogy to a nucleus.

Microörganisms abound in nature; countless myriads of them are to be found wherever there is animal or vegetable life, or decay. We ourselves swarm with them, our skins are alive, our intestinal tracts, with the exception of the stomach, and this exception has its exceptions, seems to contain more germs than anything else. And, in all probability, they are not such infrequent guests of our tissues.

The work they do and the part they play in the economy of nature should not be underrated. As a class, they do us much

---

1. Read before the Alumni Association of Niagara University, April 14, 1891.

more good than harm. They are the tireless workers in Nature's laboratory, without which we, and all animal and vegetable life, would die, for they alone prepare the food for the plant life, on which all animal life ultimately depends. They, the simplest of plant life, possess the peculiar property of not only being able to live on the higher forms of both animal and vegetable life, but in so doing to decompose their complex structures into such simpler compounds and elements as may, in their turn, serve as food for higher plants.

As to the classification of germs, it has been found convenient to divide them into —

*First.*—Moulds (Schimmelpilze), or Hypomycetes.

*Second.*—Yeasts (Hefepilze), or Blastomycetes.

*Third.*—Bacteria (Spaltpilze), Schizomycetes.

It is with the third class, the bacteria or schizomycetes, that we, as physicians, not botanists, have the most to do, and which possess for us the most interest. One word in general : we do not admit that spontaneous generation can occur ; we also hold that the various varieties of bacteria are distinct.

In classifying bacteria the first and most natural system is that based on form. It gives us (1) the bacillus, or rod-shaped cell ; (2) the coccus, or ball ; (3) the spirillum, or screw-shape — forms, which have been compared to a billiard cue, a ball and a corkscrew. It must be remembered that a bacillus is really a cylinder, and not a line ; that a coccus is really a sphere, and not flat like a griddle cake ; and a spirillum is but a twisted cylinder.

As to whether the bacteria live on dead or live substances, we divide them into saprophytes and parasites. Of the parasites it has been found convenient to make two classes : the one which cannot live and multiply outside of the animal body is called obligatory parasites ; the other, which, under favorable circumstances, can adapt itself to a life either within or outside of the animal body, we know as facultative parasites.

Disease-producing bacteria are known as pathogenic ; that they may act in either a septic (toxic) or infectious manner has long been supposed, but is now definitely known.

Bacteria multiply by fission with the most surprising rapidity. Flügge observed complete division in cocci in twenty minutes ; and Cohn estimated that, did it take an hour for complete division and for the new cells to attain maturity, one coccus would, at the end of the first day, have produced sixteen and a half millions ; at the end of the second day, 281 billions; and at end of the third day, forty-seven trillions, and at the end of the fifth day the number would be so great that all the oceans of the globe, did they have a uniform depth of one mile, would be filled.

Bacilli also germinate by means of sporulation; this process is not known to occur in the cocci and spirilli. By it we mean the formation of small, more or less round, highly refracting bodies in the bacilli. They are probably to be regarded as the seed of the plant; certainly like grains of wheat they retain the power of germination an almost indefinite period. What interests us most is the part these bacteria play, and how they play it, in the causation of disease.

We can imagine three possibilities:

*First.*—By appropriating, for their own growth, sufficient nutrient material to starve the tissues.

*Second.*—By their large number mechanically blocking the vessels.

*Third.*—By producing in the process of development, from the complex albuminoids in which they live, other and toxic substances, which, becoming absorbed, act on the general system.

It is especially the latter view to which we are leaning more and more, and already a long list of these so-called ptomaines, or tox-albumins, have been isolated, some of which we know as *tetanine*, *cadavarine*, *neurine*, *muscarine*, *mythelotoxin*, *tyrotoxicon.*

Recent experiments have settled beyond a doubt that not only the systemic effects of certain bacteria are due to the ptomaines they generate, but that the local effects are likewise dependent upon the same cause.

And still more recent experiments have shown that acquired immunity, and immunity due to inoculation with attenuated cultures, are intimately associated with the same substances.

Other investigators have demonstrated that bacteria, even pathogenic ones, are not such infrequent inhabitants of the normal tissues. As to how these bacteria are disposed of—for they, like those introduced by inoculation, soon disappear, and need not produce any symptoms, much less the disease of which they may be the cause—the theory of Metchnikoff or phagocytosis, has found rapid and widespread approbation. According to it, the leucocytes, and cells of the adenoid tissue possess the peculiar property of ingesting and digesting bacteria. Thus, it would seem, there is a constant fight going on between the phagocytes and the bacteria. If the bacteria are overcome, devoured by their enemies, the tissues continue in a healthy state; if the bacteria are victorious, then disease results.

The probability is that the leucocytes play the part of buriers of the dead rather than soldiers; and should a live bacterium be swallowed by mistake, the life of the cell pays the forfeit.

The more probable explanation is that of Petruschky, who has shown that the normal blood serum, deprived of all white cells, possesses the property of killing bacteria in limited number; that those animals which are congenitally immune to certain diseases, such as white mice and rats to anthrax, have a blood serum which possesses this property in a marked degree. He has further shown that these animals and others, such as rabbits to the staphylococcus albus, may be rendered temporarily susceptible by altering the character of their blood serum; thus, white mice and rats lose their immunity to anthrax when the alkalinity of their blood serum is diminished, and rabbits become susceptible to the staphylococcus albus when a condition of diabetes is induced.

Bacteria, according to Wyssokowitsch, are not eliminated by the secretory organs.

In the production of disease by bacteria, much depends not alone on the specific character of the germs, but on their individual virulency, on their number and on their position in the tissues, as well as on the resistance which the tissues offer; on the temperature and on the barometer.

Thus, Watson Cheyne found that in rabbits, to produce a rapidly fatal result, he must inject about one billion pus cocci, while 250 millions caused only a small abscess. With the tetanus bacillus, also on rabbits, 1,000 were necessary to produce death.

As to the virulence of the bacteria, the well-known experiments of Pasteur have familiarized us with the attenuated viruses or vaccines. We know, further, that in the abnormal, hot-house growth which we obtain of bacteria in our culture media, a diminution in the virulence is far from uncommon, also that various physical conditions as well as the chemical composition of the media may bring about this result, but it is only of late that we are beginning to understand why this is so. That it is due to the formation of toxalbumins, ptomaines, which so modify the growth as to, more or less completely, cause the pathogenic variety to once more assume the harmless habits of the saphrophyte, from which it must at some time or other have departed.

As to position in the tissues, those floating in the blood do less harm than those lodged in the tissues.

The effects of inflammation of a low grade has been shown by Max Schüller, and very recently by Watson Cheyne. They, after inoculating animals with tubercle bacilli, produced slight subcutaneous injuries, such as contusions and sprains, and found that localization generally occurred at the injured part.

Rosenberg, Hocker, Becker, Krämer and Cheyne obtained about the same result in producing acute osteomyelitis with the pus formers.

### THE RELATION OF BACTERIA TO THE FORMATION OF PUS.

The mycotic origin of pus was definitely settled by Ogsten in 1881, though Klebs years before, had come to the same conclusion.

Today we recognize as pus formers—

*First.—Staphylococcus pyogenes aureus.*

*Second.—Staphylococcus pyogenes albus.*

*Third.—Staphylococcus pyogenes citreus.*

*Fourth.—Streptococcus pyogenes.*

*Fifth.—Bacillus pyogenes fetidus.*

*Sixth.—Gonococcus of Neisser.*

*Seventh.—Micrococcus tetragenus.*

And as rarer forms the *staphylococcus pyogenes cereus albus* and *cereus flavus*, and *staphylococcus pyogenes flavescens*. By some observers the *bacillus pyocyaneus* is also regarded as a pus former.

The *bacillus tuberculosis* does *not* belong to this class. Of these, by far the most frequently met with are the *staphylococci aureus* and *albus*, the *streptococcus pyogenes* and the *gonococcus*.

The question immediately arises : Can the formation of pus be caused by other substances than the foregoing microörganisms ?

There seems to be but two possible ways in which this might occur ; either from other microörganisms as yet unknown or not settled as having this pus-forming property or from chemicals.

That pus may be formed by chemical action on the tissues has been determined : thus, croton oil, metallic mercury, or turpentine, rendered sterile, placed in sterilized glass tears, and these by an aseptic operation made to heal in under the skin of animals, will there remain inactive as long as the tear is unbroken; but, however, on breaking the tear, pus is formed, in small quantities it is true, yet, chemically and microscopically, pus. Clinically, this pus can hardly be said to occur, for it is a sterile pus, does not contain a single microörganism, and does not tend to invade the tissues.

Grawitz and de Bary have gone one step further, and have produced pus by the subcutaneous application of the toxalbumins derived from the pus formers themselves. The ptomaines were obtained from pure cultures and rendered perfectly sterile.

This, of course, is but another example of chemical irritants producing pus, but is of much more significance than the action of croton oil, for it explains how the pus formers do their work. The microörganisms generate the ptomaines, and the ptomaines by their chemical action on the tissues cause the formation of the pus.

That the microörganisms are the true and specific cause of suppuration, is proven beyond a doubt. The chain of evidence is complete. All infectious pus contains bacteria which have been cultivated, through many generations, outside the body, in pure cultures, and on inoculation with these pure cultures pus has again been formed, and the same germs found in the pus so formed.

On man these inoculations have been performed by Garré, who rubbed a quantity of a pure culture of the staphylococcus aureus into the skin of his forearm and it took weeks for the resulting carbuncle to heal.

Some inoculation experiments have failed, owing to some error in the technique, using too small a number of germs, or those which from abnormal conditions in which they are grown outside the body, had lost their virulence.

At other times abscesses have been found which contained none of the pus formers. These are almost invariably "tubercular abscesses;" when not, it is highly probable that a portion of the pus was selected in which all the germs had died—killed by their own secretions.

As a rule, the infections due to the *streptococcus pyogenes* are more serious than those due to the *staphylococcus*. This is probably because the former tends to spread along the lymph spaces, to involve more area and show less tendency to become limited.

### RELATIONS OF BACTERIA TO DIPHTHERIA.

Loeffler found in the membrane of diphtheria, but absent in the blood and tissues, a bacillus. This bacillus, he maintained, occurred regularly in every case of diphtheria, and this statement has been substantiated by Babes, Kolisko, Paltauf and others.

Loeffler himself was very reserved about declaring his bacillus as the specific cause of diphtheria, but more recent experiments by Roux and Yersin have settled the question definitely; so that we may now regard the bacillus of Loeffler as the true cause of diphtheria in man.

· The bacilli have about the average length of tubercle bacilli, but are nearly twice as broad. The form, however, is subject to variations, such as thickening of one end, transverse markings, etc.

Loeffler's bacillus does not form spores.

Cultures obtained from diphtheritic membranes do not, at first, grow well on our culture media, but seem in the course of three or four generations to become accustomed to their new food and then grow luxuriantly, though they at the same time lose their virulence.

Inoculation experiments on man have not as yet been made. On animals they soon developed the fact that not a few species possess immunity. Mice and rats will not become infected, while most birds, especially pigeons and chickens, and usually rabbits and guinea pigs, are susceptible.

On rabbits the inoculation experiments are especially interesting. They are ill for days and even weeks, thus giving time for the development of the general symptoms, the occurrence of paralysis.

The purely local occurrence of the bacilli and the severe general symptoms are best explained on the hypothesis that the bacilli, in their growth, generate a soluble substance capable of producing the systemic disturbance.

Roux and Yersin found that the sterile filtrate made from pure cultures, which were not too young, possessed the power of producing in animals, when injected subcutaneously or into the blood current, the same symptoms as do inoculations with pure cultures. No membrane occurs on the mucous surfaces, but the paralyses do occur and are usually fatal.

The larger the dose the more rapidly death ensues, but when small doses are employed, then the paralyses follow only after the lapse of many days or even weeks.

The diphtheria ptomaine is a modified albumin derived from the normal albumin of the tissues. It must, however, be noted that, clinically, the bacilli of diphtheria never occur alone, but are almost invariably associated with *streptococci*. This mixed infection is certainly important, and many observers maintain that especially fatal cases owe their virulence to the associated microörganisms.

